# The neurogenic bladder: medical treatment

**DOI:** 10.1007/s00467-007-0691-z

**Published:** 2008-05-01

**Authors:** Carla Verpoorten, Gunnar M. Buyse

**Affiliations:** Department of Child Neurology, University Hospitals K.U. Leuven, Herestraat 49, B-3000 Leuven, Belgium

**Keywords:** Neurogenic bladder, Urinary continence, Anticholinergics, Intermittent catheterization, Urodynamics

## Abstract

Neurogenic bladder sphincter dysfunction (NBSD) can cause severe and irreversible renal damage and bladder-wall destruction years before incontinence becomes an issue. Therefore, the first step in adequate management is to recognize early the bladder at risk for upper- and lower-tract deterioration and to start adequate medical treatment proactively. Clean intermittent catheterization combined with anticholinergics (oral or intravesical) is the standard therapy for NBSD. Early institution of such treatment can prevent both renal damage and secondary bladder-wall changes, thereby potentially improving long-term outcomes. In children with severe side effects or with insufficient suppression of detrusor overactivity despite maximal dosage of oral oxybutynin, intravesical instillation is an effective alternative. Intravesical instillation eliminates systemic side effects by reducing the first-pass metabolism and, compared with oral oxybutynin, intravesical oxybutynin is a more potent and long-acting detrusor suppressor. There is growing evidence that with early adequate treatment, kidneys are saved and normal bladder growth can be achieved in children so they will no longer need surgical bladder augmentation to achieve safe urinary continence in adolescence and adulthood.

## Introduction

Neurogenic bladder sphincter dysfunction (NBSD) can develop as a result of a lesion at any level in the nervous system, including the cerebral cortex, spinal cord, or peripheral nervous system. Neurologic conditions in children leading to neurogenic bladder dysfunction are predominantly congenital neural tube defects (including myelomeningocele, lipomeningocele, sacral agenesis, and occult lesions causing tethered cord). Acquired causes such as spinal cord tumors or trauma or sequelae of transverse myelitis are less frequent. Whereas from an etiologic standpoint neurogenic bladder dysfunction is a heterogeneous group, medical management will be similar irrespective of the underlying cause. The vast majority of knowledge about NBSD management comes from long-term experience with myelomeningocele (MMC), the most common neural tube defect.

Following the institution of a general treatment policy with advances in neurosurgical and orthopedic treatments in previous decades, governing the associated NBSD has become crucial for improving quality of life and life expectancy in children with neural tube defects. In MMC patients, disordered innervation of the detrusor musculature and external sphincter adversely affects bladder function, which if untreated not only leads to incontinence but also will cause secondary damage and dysfunction of both the upper and lower urinary tracts. Key elements in optimal NBSD management are early diagnosis (including NBSD typology) and early (presymptomatic) institution of adequate medical treatment. There is indeed growing evidence that management decisions made during infancy, which prevent both renal damage and secondary bladder-wall changes, potentially impact long-term outcomes for renal function and safe urinary continence.

After describing the pathophysiology of NBSD and its possible consequences, this paper focuses on diagnosis (including early identification of patients at risk), treatment goals, treatment tools, and practical management of NBSD.

## Historical evolution

The management of NBSD in children has undergone major changes over the years. A first milestone was the introduction of clean intermittent catheterization (CIC) in 1972 [1]. CIC (combined with anticholinergics if required) has made “conservative” (medical) management a successful treatment option, with a good outcome for quality of life and kidney protection. Further important breakthroughs were the wider application of urodynamic testing in the evaluation of infants and young children with suspected NBSD [2–4] and the better pathophysiological understanding of the natural history of NBSD in patients with spina bifida. In spina bifida, the natural history of the urinary tract in untreated NBSD is one of progressive deterioration by the age of 3 years in up to 58% of patients [5]. Several reports have shown this deterioration to be directly related to increased intravesical pressure. In 1981, the bladder pressure at which urethral leakage occurred was found to be a useful predictor of unsafe bladder function [2]. The leak-point pressure, as it is now commonly referred to, has become accepted as one of the urodynamic parameters that allows clinicians to differentiate patients with relatively low or high risk for subsequent upper urinary tract deterioration. In 1984, detrusor external sphincter dyssynergia (DSD) was identified as an important factor leading to functional obstruction, and intravesical pressure was recognized as the pathophysiological mechanism of subsequent upper urinary tract deterioration [3]. Shortly thereafter, urodynamics in infants and children was shown to allow a functional classification of NBSD that correlated with clinical entities of incontinence and obstruction, an approach that has allowed the concept of individualized and presymptomatic therapy in high-risk patients [6].

## Pathophysiology of the neurogenic bladder

Under normal conditions, the detrusor muscle, bladder neck, and striated external sphincter function as a synergistic unit for adequate storage and complete evacuation of urine. In healthy bladders, the change in bladder-filling pressure between empty and full is normally less than 10–15 cm H_2_O. Normal voiding pressures for males and females are from 50 to 80 cm H_2_O and from 40 to 65 cm H_2_O, respectively [7].

In patients with NBSD, disordered innervation of the detrusor musculature and external sphincter adversely affects bladder function. In recent years, it has become clear that children with this condition can be categorized into high- and low-risk groups for secondary damage from a neurogenic bladder based on intravesical pressure. When the detrusor (filling) pressure exceeds 40 cm H_2_O, glomerular filtration rate decreases and pyelocaliceal and ureteral drainage deteriorates, leading to obstructive hydronephrosis and/or vesicoureteral reflux [2, 8–10]. Even in the absence of reflux or upper urinary tract dilatation, high intravesical pressure can impair drainage of urine into the bladder. Any pathophysiologic process that causes either intermittent or continuous elevation of bladder pressure above 40 cm H_2_O places the child at risk for upper urinary tract dysfunction, urinary tract infections, and ultimately renal failure. Intermittent elevation of bladder pressure may occur from detrusor hypertonia, hyperreflexia, or both. Hyperreflexia may cause intermittent elevation of bladder pressure, especially if the external sphincter acts reflexively and tightens rather than relaxes in an attempt to prevent micturition [detrusor sphincter dyssynergia (DSD)]. Over a long period of time, hyperreflexia with pressures greater than 40 cm H_2_O may result in detrusor decompensation (areflexia from myogenic failure) or in detrusor hypertrophy with associated sacculations and subsequent diverticula formation. These pathophysiologic changes affect the elastic and vesicoelastic properties of the bladder and also result in mechanical ureterovesical junction obstruction. Continuous elevation of bladder pressure above 40 cm H_2_O may occur from a hypertonic detrusor or a hypertrophic small-capacity bladder secondary to outflow obstruction [11]. Bladder outlet obstruction is caused by DSD, or by fibrosis of the external urethral sphincter secondary to partial or complete denervation [3, 12, 13]. Bladder outlet obstruction will lead to elevated (pathologic) voiding pressures, which will contribute to either detrusor decompensation or hypertrophy. Finally, recurrent urinary tract infections due to bladder residue may aggravate damage to the neurogenic bladder through processes of transmural inflammation and fibrosis. Together with high intravesical pressures and/or vesicoureteral reflux, these lower urinary tract infections will lead to episodes of acute pyelonephritis and irreversible renal damage.

## Management of the neurogenic bladder

### General principles and treatment goals

The cornerstone of optimal NBSD management is early identification and characterization (typology) and the institution of proactive therapy. Crucial for long-term prognosis of patients with NBSD is the fact that the management must start before consequences of bladder dysfunction become apparent. From the outset, the goals of management are to prevent or minimize secondary damage to the upper urinary tracts and bladder from the primary neurogenic bladder dysfunction and to achieve safe social continence [14]. Thus, long before continence becomes an issue, starting from the first year of life, management is directed at creating a low-pressure reservoir and ensuring complete and safe bladder emptying.

Clean intermittent catheterization (CIC) or self-catheterization (CISC) in combination with anticholinergics (oxybutynin) is the standard therapy for children with neurogenic bladder dysfunction with detrusor hyperactivity and/or DSD [11, 15, 16]. This treatment is also feasible and effective in developing countries, where untreated neuropathic bladder is an important cause of preventable chronic renal failure [17, 18]. CIC enables complete bladder emptying and thus avoids bladder residues and consequent risks for infections. In the high-risk bladder with DSD, CIC also allows bladder emptying before the occurrence of otherwise “spontaneous” high-pressure voiding, which is known to be detrimental for kidney function and drainage. Oxybutynin, a bladder smooth-muscle relaxant, is used to improve bladder dynamics through suppression of detrusor hypertonicity and hyperreflexia. By doing so, oxybutynin eliminates (high-pressure) uninhibited detrusor contractions (and thus urinary leakage) and prevents high-pressure bladder storage (due to detrusor hypertonicity or low bladder compliance) and high-pressure emptying (in case of DSD).

### Early management, including diagnosis and identification of the high-risk bladder

At birth, the majority of patients with neurogenic bladder has normal upper urinary tracts. Without proper management, urinary tract infections and elevated bladder pressures with secondary bladder-wall changes may cause upper urinary tract deterioration within 3 years in up to 58% [5]. One third of children who develop impaired kidney drainage do so within the first year of life [19]. The specific abnormalities vary considerably and are not predicted by the level of the spinal cord defect. Furthermore, the dysfunctional pattern may be dynamic, influenced by spinal cord surgery, tethering, and denervation. In the initial baseline evaluation, clinical observations must be completed with urinalysis (microscopy and culture), renal/bladder ultrasound, and cystourethrogram. These allow the experienced clinician to suspect the type of NBSD and to identify the high-risk subgroup. The next consideration is when to perform urodynamic studies.

Two different opinions exist in the literature on the use of urodynamic studies in the early evaluation and further follow-up. In one approach, urodynamic assessment has become an integral part of the initial evaluation and subsequent management, as it allows recognition of the different subtypes of NBSD (typology), proactive interventions, evaluation and guidance of therapy, and early detection of neurologic deterioration (such as symptomatic tethering of the spinal cord [20]). Advocates justify this approach of routine urodynamics to minimize the deleterious effects of high intravesical pressure by directly measuring it rather than indirectly suspecting it from the development of upper and lower urinary tract changes on serial radiologic imaging. Several studies have shown that early urodynamic evaluation of children with NBSD allows the prediction of which newborns are at risk for upper urinary tract deterioration. Urodynamic risk factors are low bladder compliance, intravesical pressure more than 40 cm H_2_O, and DSD [2, 3, 6, 21]. The alternative to urodynamic-based management is serial radiologic imaging to detect secondary evidence of high bladder pressure. Critics of newborn and early infancy urodynamics refer to a lack of standards for performance and interpretation, which might lead to unnecessary interventions [22, 23]. Those authors recommend careful history, physical examination, upper urinary tract imaging, and close follow-up during infancy and childhood, reserving urodynamic studies only for patients with evidence of urinary retention on physical examination, new-onset hydronephrosis or febrile urinary tract infection, or for evaluation to achieve continence. Proponents of this approach with selective urodynamics suggest that close monitoring with prompt intervention at first signs of deterioration is effective in protecting the upper urinary tracts (including preservation of nephrons and thus renal function in the long run). A remaining concern, however, could be that in this more expectant approach, high intravesical pressures may have already resulted in irreversible and avoidable damage to the bladder wall, resulting in small-capacity, low-compliance bladders later in life.

Although many questions regarding optimal evaluation and management remain unanswered, the consensus on the need of close surveillance, especially in the first years of life, plus the possibility that proactive treatment may be better for the bladder in the very long term, emphasize the need for an integrated approach in which clinical observations, serial imaging, and urodynamics are the basis for early adequate treatment.

### Urodynamic studies: special considerations in children with NBSD

If properly performed, even with possible shortcomings in newborns and infancy, urodynamic studies allow direct diagnosis of NBSD and recognition of dysfunction subtypes. This functional classification allows adequate treatment for the different types and early proactive treatment for the bladder at risk [6].

It is important for the practitioner to understand the complexities involved in performing urodynamic studies in newborns, infants, and children. Urodynamic assessment can provide reproducible results in newborns and infants, but it requires attention to mechanical factors and filling rates. The younger the child, the higher the risk that mechanical factors (such as bladder-outlet obstruction by the catheter used for the investigation) may produce artificial information (elevated leak pressure or inability to void). It has also been shown that using a bladder infusion rate as close as possible to the natural filling rate is important for correct assessment of detrusor properties [24]. It is presumed that fast infusion rates overcome vesicoelastic detrusor properties, falsely indicating detrusor hypertonicity [11]. On the other hand, in children who have apparent low-pressure cystograms and who leak during filling (due to sphincter hypoactivity), detrusor hypertonia may be unrecognized [25]. In these children, it is important to perform a provocative study (including bladder neck occlusion with a balloon catheter) to identify unrecognized detrusor hyperactivity prior to bladder-neck surgery for treatment of incontinence. Electromyographic (EMG) evaluation of the external urethral sphincter is required to identify DSD. The use of concentric EMG needles is preferred, as it gives more reliable information than patch electrodes [11]. The combination of X-ray cystography with cystometrogram and sphincter EMG (video urodynamics) allows accurate evaluation of the link between intravesical pressure and vesicoureteral reflux and gives direct visual information of (dys)synergia between detrusor and sphincter mechanisms [26].

### Clean intermittent catheterisation

In children with neurogenic bladder, CIC is the first-choice treatment to empty the bladder adequately (no residue, no infection) and safely (prior to high-pressure voiding), and it is a valuable tool for achieving continence. The wide variety of used materials and techniques for CIC does not seem to affect efficacy and safety as long as some basic principles are applied: proper education and training, clean and atraumatic application, and achievement of good patient compliance on a long-term basis. For education, training, and further guidance during follow-up, a dedicated continence nurse is invaluable. Patients and caregivers must understand what is wrong with the bladder/sphincter and why CIC is proposed for treatment, and they have to learn how to catheterize properly. CIC has been successfully used by parents even in newborns and infants, becoming a part of their everyday routine [27]. Some authors prefer early institution of CIC in all infants with NBSD, given the fact that by the age of 3 years, CIC will be required in all for achieving continence, and given the difficulties of starting CIC at toddler age [28]. Such early institution of CIC seems to improve family compliance and their ability to assist the child in coping with their disease and with CIC [29]. CISC can be successfully taught to boys and girls who are motivated and who have developed the required dexterity, mostly around the age of 6 years. The required frequency of catheterization depends on several factors: fluid intake, bladder capacity, and bladder filling/voiding pressures. In practice, it is recommended to catheterize six times a day in infants (linked with feeding time) and five times a day in school-aged children. Although reported incidences of CIC-related infection risks are variable, it is generally agreed that the risk is low as long as complete bladder emptying is achieved. Furthermore, reused supplies are not related to more urinary tract infections [30]. If symptomatic infections occur, these are mainly caused by incomplete bladder emptying, and CIC appliance by child or caregiver needs to be optimized. To prevent urethral strictures and false passage in boys, catheter lubrication and avoidance of forceful manipulation during catheter insertion are advocated. Nonreusable low-friction catheters are considered valuable in high-risk male patients with urethral false passage or very tense sphincters but are unnecessary in routine cases [31]. To maintain therapeutic compliance with CISC in adolescents, psychosocial support is often required. Neurogenic bowel dysfunction with constipation and fecal soiling can interfere with the institution of a successful CIC treatment. Retained stools may mechanically impair bladder filling, increase detrusor irritability, or contribute to urine retention. Stool incontinence increases the risk of bladder contamination and urinary tract infection. An effective bowel management program is therefore needed. Finally, given the high prevalence of latex allergy [32], in the spina bifida population, a strict latex-free approach is of extreme importance.

### Pharmacologic treatment: anticholinergics

Of the anticholinergic agents available, oxybutynin hydrochloride is most commonly used, and long-term experience supports its safety also in newborns and infants [33]. Oxybutynin is a tertiary amine with a well-documented therapeutic effect on detrusor hyperactivity, and its effectiveness is attributed to a combination of anticholinergic (M3-selective receptor subtype antagonism), antispasmodic, local anesthetic and calcium-channel-blocking activity [34]. Several studies have shown its efficacy for decreasing the filling pressure, increasing the capacity of the neurogenic bladder, and preserving renal function [35–37]. The usual dose regimen of oral oxybutynin is 0.3–0.6 mg/kg per day in three doses.

In children with insufficient response or significant systemic side effects to oral oxybutynin, intravesical instillation of oxybutynin has been shown to be a highly efficacious, reliable, and well-tolerated therapy for children who would otherwise require surgical therapy [38–43]. Because a solution suitable for intravesical instillation was not available, crushed oxybutynin tablets were used in the earlier trials, with consequent problems of inconvenience and impracticability, and it was the belief of several authors that poor patient compliance could be resolved by an optimized drug preparation [40, 44]. It was subsequently shown that, indeed, eliminating the complex crushing preparation by child or parent makes intravesical oxybutynin therapy easy to use and acceptable for long-term therapy [41].

The mechanisms underlying the more potent and longer-acting detrusor-suppressive effects of intravesical oxybutyinin, as well as its better tolerability, have been investigated by several groups. It was demonstrated that a reduced first-pass metabolism of oxybutynin after intravesical instillation, resulting in a reduced generation of the N-desethyl metabolite, may explain the clinically relevant reduction of systemic side effects that characterizes intravesical compared with oral oxybutynin therapy [45]. In addition, these pharmacokinetic studies provided first evidence for a direct local rather than a systemic effect of intravesical oxybutynin on detrusor muscle [45]. Further evidence for a local effect of intravesically administered oxybutynin was provided by studies showing local (urothelial) accumulation, suppression of muscarinic receptor-mediated detrusor muscle contractions, and blocking of muscarinic receptors in bladder-afferent pathways [46, 47]. In most reports, intravesical oxybutynin is used in dosages between 0.3 and 0.6 mg/kg per day in two or three doses. Given its better tolerability compared with oral treatment, if required, intravesical dosages can be further increased up to doses of 0.9 mg/kg per day [43].

To date, the vast majority (∼ 90%) of patients can be treated successfully with the gold standard treatment of oxybutynin (oral or intravesical) and CIC. Other bladder-relaxant drugs include propiverine (10–15 mg b.i.d. or t.i.d., adult dose), trospium (20 mg b.i.d., adult dose), extended-release oxybutynin, and tolterodine (children 0.25–1 mg b.i.d., adults 1–2 mg b.i.d.). The current experience with compounds other than oxybutynin is still limited in children with neurogenic bladder [48, 49]. Botulinum A toxin injections into the detrusor muscle have been shown to be a potentially valuable approach in the neurogenic overactive bladder [50]. Repeated botulinum A toxin injections (as an alternative for or an additive to anticholinergics) could be considered to postpone or avoid surgical procedures in the small minority of children not responding to standard therapy with CIC and anticholinergics [51]. However, further investigations are required, given remaining concerns about costs and long-term efficacy and safety of prolonged botulinum A toxin administration. Although some authors have advocated alfa-receptor stimulation of the bladder neck, no validated medical treatment is available to enhance the bladder outlet.

### Medical management of NBSD in clinical practice

Optimal management involves first, early diagnosis, including recognition of high-risk subtypes, and second, proactive institution of adequate treatment. Early proactive treatment of high-pressure dyssynergic lower urinary tracts is important in the long term, not only to preserve renal function [52] but also to prevent poor bladder compliance and the subsequent need for bladder augmentation [35]. Urodynamic assessment is used in newborns and infants to come to a functional classification of NBSD, allowing presymptomatic interventions in the high-risk groups and individualized treatment planning according to the type of dysfunction [6, 29, 53].

**Fig. 1:**
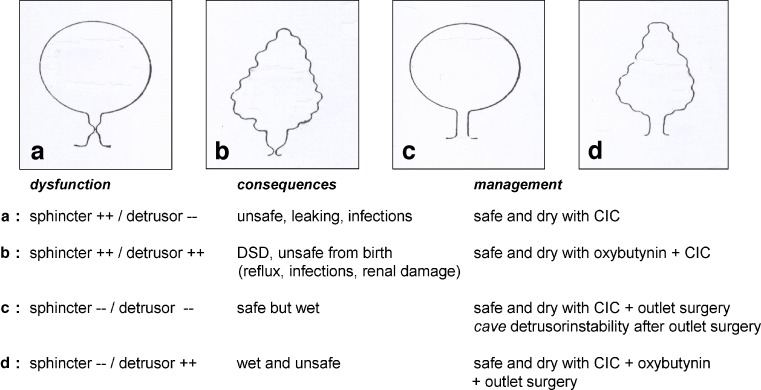
Classification of the neurogenic bladder, with four subtypes (**a–d**) according to dysfunctional activities of sphincter and detrusor. For each subtype, clinical implications if untreated and principles of management are summarized

In clinical practice, four major subtypes can be used to describe NBSD (Fig. 1): sphincter overactivity combined with detrusor underactivity (type A) or overactivity (type B), and sphincter underactivity combined with detrusor underactivity (type C) or with detrusor overactivity (type D). The easiest type to treat is type A. This bladder type requires early treatment because of urine retention with high filling pressure and continuous leaking. Here, CIC alone is effective and sufficient and will make the bladder safe and infection free, and the patient will be dry in between (social continence). Good care to empty the bladder totally is most important to avoid bladder infections caused by residual urine. Dysfunctional type B will have high filling and high voiding pressures, being very unsafe from birth onward due to DSD. Here, the act of voiding has to be prevented. With oxybutynin, the overactive detrusor can be “pharmacologically converted” to an inactive reservoir (situation similar to type A), which has to be emptied with CIC. In type C, CIC reduces the degree of incontinence and offers much better control over urinary tract infections. To achieve continence, this type will at a later age need surgical intervention on the sphincter (e.g. sling operation). An important caveat here is that detrusor instability may emerge only after surgical improvement of outlet resistance. If this detrusor instability would remain unrecognized and untreated (with oxybutynin), bladder-outlet surgery would have converted a “wet but safe” into a “dry but unsafe” bladder. In the last dysfunctional subtype (type D), the bladder leaks due to detrusor instability and gradually becomes unsafe due to secondary bladder-wall changes with detrusor hypertrophy and loss of bladder compliance. Therefore, treatment consists of CIC combined with oxybutynin and, at a later age, bladder-outlet surgery.

Once appropriate therapy has been initiated, adequate follow-up is required, with adjustments if needed (CIC frequency, medication dosing and administration route). Treatment efficacy can be assessed using clinical parameters (including CIC frequency and volume charts), urinalysis, renal and bladder ultrasound, X-ray cystography, and video urodynamics.

As long-term sequelae of insufficiently treated neurogenic bladders (renal scarring, noncompliant fibrotic bladder) already have their origin in the first years of life, the frequency of multidisciplinary follow-up visits must be age dependent (3× yearly up to age 3 years, 2× yearly in school-aged children, yearly in adults). Typically, urinalysis and ultrasound are performed at all visits, cystography to investigate unexpected upper urinary tract infections, and urodynamics periodically to verify that under treatment, the catheterized bladder volumes are age appropriate [54] and stored under safe pressure conditions (storage of expected bladder capacity at pressures below 30 cm H_2_O; see [55]).

**Fig. 2:**
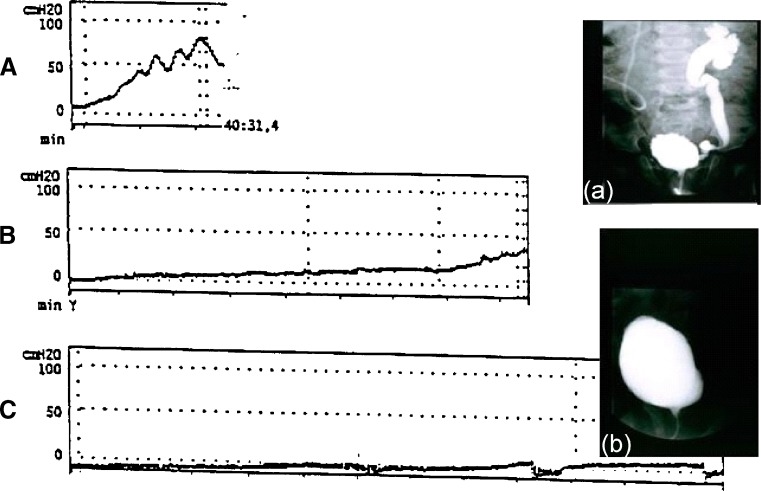
Suppression of detrusor hyperactivity with resolution of reflux by nonsurgical management. Illustrative patient with high-risk neurogenic bladder sphincter dysfunction (NBSD) (type B), urodynamically showing early unsafe filling pressures (**A**) with high-grade reflux (**a**) and urosepsis before treatment. Under clean intermittent catheterization (CIC) plus oxybutynin, the unsafe high-pressure bladder was converted into a safe low-pressure reservoir with good capacity and disappearance of the reflux at control cystography 3 months later (**b**). Severe systemic side effects, making continuation of oral oxybutynin impossible, disappeared after switching to intravesical oxybutynin. Further urodynamic evaluations (**B**: after first intravesical administration; **C**: after 4 months) documented adequate suppression of detrusor hyperactivity (modified from [40]). Long-term (currently 13 years) continuation of CIC and intravesical oxybutynin has resulted in a safe and adequate capacity bladder with social continence for the patient

With early instituted and optimal treatment, the large majority of patients can be adequately controlled without antireflux surgery or surgical bladder augmentation (Fig. 2). Augmentation cystoplasty is limited to a small group of patients in whom medical treatment fails (persistence of high filling pressures). In patients with insufficient sphincter activity, continence achievement will require bladder-outlet surgery in addition to medical treatment. In female wheelchair users, surgical intervention to provide a continent stoma will facilitate self-catheterization.

### Long-term outcome evaluation and need for life-long follow-up

Lifelong follow-up with further periodic investigations of upper urinary tract changes, renal function, and bladder status is extremely important. There are two reasons why long-term outcome evaluation in adulthood and life-long patient follow-up are indispensable. First, for the individual patient, therapy is a life-long requisite, and verifying preservation of the patient’s kidneys is only possible by repetitive assessment throughout adolescence and adulthood. Second, in general, detailed long-term follow-up data will show whether a treatment policy driven by long-term goals is sufficiently effective or requires further adaptations. The effectiveness of efforts preserving upper urinary tract function can only be judged by assessing the ultimate outcome once these patients have reached adolescence or adulthood [29]. In populations with NBSD, no consensus exists as to how renal status is ideally evaluated [56]. In clinical practice, upper urinary tract deterioration or protection is often monitored by radiographic images of hydronephrosis and vesicoureteral reflux. Modalities used to look at renal functions include nuclear imaging [dimercaptosuccinate acid (DMSA) renal scan], urinary concentrating ability, and glomerular filtration rate assessment. For the latter, creatinine (Cr) clearance can be used for patients who are socially continent; for others, inulin or Cr ethylenediaminetetraacetate (EDTA) clearance can be used. Which (combination) of these tests is best to evaluate renal function requires further investigation [56].

## Conclusions

Medical management with CIC and anticholinergics is effective in preserving renal function and providing safe urinary continence in more than 90% of patients with a neurogenic bladder. Early diagnosis and treatment institution, long before continence becomes an issue at toddler age, can prevent both renal damage and secondary bladder-wall changes, thereby improving long-term outcomes. Compared with oral oxybutynin, intravesical oxybutynin has more potent and longer-acting detrusor suppressive effects with good tolerance and should be used prior to considering surgical therapies. Therapeutic goals should no longer be restricted to prevention of secondary damage to both upper and lower urinary tracts. Instead, our goal should be to achieve normal renal and bladder growth at safe bladder pressure, with appliance-free continence.

## Study questions

(Answers appear following the reference list)

Directions: Each of the numbered questions is followed by several possible answers.

Select the one lettered answer that is *NOT CORRECT* in each case.

1. Medical management of NBSD will be similar, irrespective of :
  - Underlying cause
  - Patient age
  - Pattern of bladder dysfunction
2. Depending on the pattern of bladder dysfunction, safe urinary continence can be achieved by:
  - CIC + anticholinergics + bladder-neck surgery
  - Bladder-neck surgery alone
  - CIC alone
3. Urodynamic risk factors for upper urinary tract deterioration are:
  - DSD
  - High bladder compliance
  - Intravesical pressure >40 cm H_2_O
4. In a patient where CIC was started and in which catheterized volumes are always far below the expected bladder capacity for age:
  - There is no indication for CIC, and CIC can be stopped
  - Anticholinergic treatment may need to be added to CIC
  - Bladder-outlet resistance may be very insufficient
5. In up to 90% of patients, optimal medical NBSD management can prevent:
  - Vesicoureteral reflux surgery
  - Surgical augmentation cystoplasty
  - Bladder-outlet surgery

## Abbreviations

NBSD: neurogenic bladder sphincter dysfunction
MMC: myelomeningocele
CIC: clean intermittent catheterization
CISC: clean intermittent self-catheterization
DSD: detrusor sphincter dyssynergia

## References

[1] Lapides J, Diokno AC, Silber SJ, Lowe BS (1972). Clean intermittent self-catheterization in the treatment of urinary tract disease. J Urol.

[2] McGuire EJ, Woodside JR, Bordin TA, Weiss RM (1981). Prognostic value of urodynamic testing in myelodysplastic patients. J Urol.

[3] Bauer SB, Hallet M, Khoshbin S, Lebowitz RL, Winston KR, Gibson S, Colodny AH, Retik AB (1984). Predictive value of urodynamic evaluation in newborns with myelodysplasia. JAMA.

[4] Sidi AA, Peng W, Gonzalez R (1986). Vesicoureteral reflux in children with myelodysplasia: natural history and results of treatment. J Urol.

[5] Smith ED (1972). Urinary prognosis in spina bifida. J Urol.

[6] van Gool JD (1986). Spina bifida and neurogenic bladder dysfunction—a urodynamic study.

[7] Stephenson TP, Wein AJ, Mundy AR, Stephenson TP, Wein A (1986). The interpretation of urodynamics. Urodynamics: principles, practice and application.

[8] Wang SC, McGuire EJ, Bloom DA (1988). A bladder pressure management system for myelodysplasia–clinical outcome. J Urol.

[9] Steinhardt GF, Goodgold HM, Samuels LD (1988). The effect of intravesical pressure on glomerular filtration rate in patients with myelomeningocele. J Urol.

[10] Rickwood AM, Thomas DG, Philp NH, Spicer RD (1982). Assessment of congenital neurovesical dysfunction by combined urodynamic and radiological studies. Br J Urol.

[11] Bauer SB, Joseph DB (1990). Management of the obstructed urinary tract associated with neurogenic bladder dysfunction. Urol Clin North Am.

[12] van Gool JD (1982). Detrusor-sphincter dyssynergia in children with myelomeningocele: a prospective study. Z Kinderchir.

[13] Mundy AR, Borzyskowski M, Saxton HM (1982). Videourodynamic evaluation of neuropathic vesicoureteral dysfunction in children. J Urol.

[14] Snodgrass WT, Adams R (2004). Initial urologic management of myelomeningocele. Urol Clin North Am.

[15] Baskin LS, Kogan BA, Benard F (1990). Treatment of infants with neurogenic bladder dysfunction using anticholinergic drugs and intermittent catheterisation. Br J Urol.

[16] Fernandes E, Reinberg Y, Vernier R, Gonzales R (1994). Neurogenic bladder dysfunction in children: review of pathophysiology and current management. J Pediatr.

[17] Jeruto A, Poenaru D, Bransford R (2004). Clean intermittent catheterisation: overview of results in 194 patients with spina bifida. Afr J Pediatr Surg.

[18] Kari JA (2006). Neuropathic bladder as a cause of chronic renal failure in children in developing countries. Pediatr Nephrol.

[19] Spindel MR, Bauer SB, Dyro FM, Krarup C, Koshbin S, Winston KR, Lebowitz RL, Colodny AH, Retik AB (1987). The changing neurourologic lesion in myelodysplasia. JAMA.

[20] Tarcan T, Bauer S, Olmedo E, Khoshbin S, Kelly M, Darbey M (2001). Long-term followup of newborns with myelodysplasia and normal urodynamic findings: is follow-up necessary?. J Urol.

[21] Kurzrock EA, Polse S (1998). Renal deterioration in myelodysplastic children: urodynamic evaluation and clinical correlates. J Urol.

[22] Teichman JM, Scherz HC, Kim KD, Cho DH, Packer MG, Kaplan GW (1994). An alternative approach to myelodysplasia management: aggressive observation and prompt intervention. J Urol.

[23] Hopps CV, Kropp KA (2003). Preservation of renal function in children with myelomeningocele managed with basic newborn evaluation and close follow-up. J Urol.

[24] Joseph DB (1992). The effect of medium-fill and slow-fill saline cystometry on detrusor pressure in infants and children with myelodysplasia. J Urol.

[25] Bauer SB, Reda EF, Colodny AH, Retik AB (1986). Detrusor instability: a delayed complication in association with the artificial sphincter. J Urol.

[26] Blaivas JG, Labib KL, Bauer SB, Retik AB (1977). A new approach to electromyography of the external urethral spincter. J Urol.

[27] Joseph DB, Bauer SB, Colodny AH, Mandell J, Retik AB (1989). Clean intermittent catheterization of infants with neurogenic bladder. Pediatrics.

[28] van Gool JD, Dik P, de Jong TP (2001). Bladder sphincter dysfunction in myelomeningocele. Eur J Pediatr.

[29] Kessler TM, Lackner J, Kiss G, Rehder P, Madersbacher H (2006). Early proactive management improves upper urinary tract function and reduces the need for surgery in patients with myelomeningocele. Neurourol Urodyn.

[30] Schlager TA, Clark M, Anderson S (2001). Effects of single-use sterile catheter for each void on the frequency of bacteriuria in children with neurogenic bladder on intermittent catheterization for bladder emptying. Pediatrics.

[31] Sutherland RS, Kogan BA, Baskin LS, Mevorach RA (1996). Clean intermittent catheterization in boys using the LOFric catheter. J Urol.

[32] De Swert LF, Van Laer KM, Verpoorten CM, Van Hoeyveld EM, Cadot P, Stevens EA (1997). Determination of independent risk factors and comparative analysis of diagnostic methods for immediate type latex allergy in spina bifida patients. Clin Exp Allergy.

[33] Edelstein RA, Bauer SB, Kelly MD, Darbey MM, Peters CA, Atala A, Mandell J, Colodny AH, Retik AB (1995). The long-term urological response of neonates with myelodysplasia treated proactively with intermittent catheterization and anticholinergic therapy. J Urol.

[34] Andersson KE, Chapple CR (2001). Oxybutynin and the overactive bladder. World J Urol.

[35] Kaefer M, Pabby A, Kelly M, Darbey M, Bauer SB (1999). Improved bladder function after prophylactic treatment of the high risk neurogenic bladder in newborns with myelomeningocele. J Urol.

[36] Aslan AR, Kogan BA (2002). Conservative management in neurogenic bladder dysfunction. Curr Opin Urol.

[37] Dik P, Klijn AJ, van Gool JD, de Jong-de Vos van Steenwijk CC, de Jong TP (2006). Early start to therapy preserves kidney function in spina bifida patients. Eur Urol.

[38] Brendler CB, Radebaugh LC, Mohler JL (1989). Topical oxybutynin chloride for relaxation of dysfunctional bladders. J Urol.

[39] Madersbacher H, Jilg G (1991). Control of detrusor hyperreflexia by the intravesical instillation of oxybutynin hydrochloride. Paraplegia.

[40] Buyse G, Verpoorten C, Vereecken R, Casaer P (1995). Treatment of neurogenic bladder dysfunction in infants and children with neurospinal dysraphism with clean intermittent (self) catheterisation and optimized intravesical oxybutynin hydrochloride therapy. Eur J Pediatr Surg.

[41] Buyse G, Verpoorten C, Vereecken R, Casaer P (1998). Intravesical application of a stable oxybutynin solution improves therapeutic compliance and acceptance in children with neurogenic bladder dysfunction. J Urol.

[42] Amark P, Bussman G, Eksborg S (1998). Follow-up of long-time treatment with intravesical oxybutynin for neurogenic bladder in children. Eur Urol.

[43] Haferkamp A, Staehler G, Gerner HJ, Dörsam J (2000). Dosage escalation of intravesical oxybutynin in the treatment of neurogenic bladder patients. Spinal Cord.

[44] Fratta A, Bordenave J, Boissinot C, Le Grand J, Esquirol C, Radideau E, Benoit G (2005). Development of an intravesical oxybutynin chloride solution: from formulation to quality control. Ann Pharm Fr.

[45] Buyse G, Waldeck K, Verpoorten C, Björk H, Casaer P, Andersson KE (1998). Intravesical oxybutynin for neurogenic bladder dysfunction: less systemic side effects due to reduced first pass metabolism. J Urol.

[46] De Wachter S, Wyndaele JJ (2003). Intravesical oxybutynin: a local anesthetic effect on bladder C afferents. J Urol.

[47] Kim Y, Yoshimura N, Masuda H, de Miguel F, Chancellor MB (2005). Antimuscarinic agents exhibit local inhibitory effects on muscarinic receptors in bladder afferent pathways. Urology.

[48] Youdim K, Kogan BA (2002). Preliminary study of the safety and efficacy of extended-release oxybutynin in children. Urology.

[49] Goessl C, Sauter T, Michael T, Bergé B, Staehler M, Miller K (2000). Efficacy and tolerability of tolterodine in children with detrusor hyperreflexia. Urology.

[50] Reitz A, Stöhrer M, Kramer G, Del Popolo G, Chartier-Kastler E, Pannek J, Burgdörfer H, Göcking K, Madersbacher H, Schumacher S, Richter R, von Tobel J, Schurch B (2004). European experience of 200 cases treated with Botulinum-A toxin injections into the detrusor muscle for urinary incontinence due to neurogenic detrusor overactivity. Eur Urol.

[51] Altaweel W, Jednack R, Bilodeau C, Corcos J (2006). Repeated intradetrusor botulinum toxin type A in children with neurogenic bladder due to myelomeningocele. J Urol.

[52] Kasabian NG, Bauer SB, Dyro FM, Colodny AH, Mandell J, Retik AB (1992). The prophylactic value of clean intermittent catheterization and anticholinergic medication in newborns and infants with myelodysplasia at risk of developing urinary tract deterioration. Am J Dis Child.

[53] Madersbacher H (2002). Neurogenic bladder dysfunction in patients with myelomeningocele. Curr Opin Urol.

[54] Berger R, Maizels M, Moran G, Conway J, Firlit C (1983). Bladder capacity (ounces) equals age (years) plus 2 predicts normal bladder capacity and aids in diagnosis of abnormal voiding patterns. J Urol.

[55] Houle AM, Gilmour RF, Churchill BM, Gaumond M, Bissonnette B (1993). What volume can a child normally store in the bladder at a safe pressure?. J Urol.

[56] Liptak GS (2003) Evidence-based practice in spina bifida: developing a research agenda, May 9–10, 2003, Washington DC

